# Inhibition of DAMP signaling as an effective adjunctive treatment strategy in pneumococcal meningitis

**DOI:** 10.1186/s12974-017-0989-0

**Published:** 2017-11-02

**Authors:** Ilias Masouris, Matthias Klein, Susanne Dyckhoff, Barbara Angele, H. W. Pfister, Uwe Koedel

**Affiliations:** 1 Department of Neurology, University Hospital, LMU Munich, 81377 Munich, Germany; 2 Department of Neurology, Klinikum Grosshadern of the Ludwig Maximilians University, Marchioninistraße 15, 81377 Munich, Germany

**Keywords:** *Streptococcus pneumoniae*, Meningitis, HMGB1, Paquinimod, Adjuvant treatment

## Abstract

**Background:**

Pneumococcal meningitis remains a potentially lethal and debilitating disease, mainly due to brain damage from sustained inflammation. The release of danger-associated molecular patterns (DAMPs), like myeloid-related protein 14 (MRP14) and high mobility group box 1 protein (HMGB1), plays a major role in persistence of inflammation. In this study, we evaluated if paquinimod, an MRP14-inhibitor, and an anti-HMGB1 antibody can improve clinical outcome as adjunctive therapeutics in pneumococcal meningitis.

**Methods:**

We tested the adjuvant administration of paquinimod and the anti-HMGB1 antibody in our pneumococcal meningitis mouse model assessing clinical (clinical score, open-field-test, temperature) and pathophysiological parameters (intracranial pressure, white blood cell count in CSF, bleeding area) as well as bacterial titers in blood and brain 24 h after administration and 48 h after infection. Furthermore, we explored the interactions of these two agents with dexamethasone, the standard adjuvant treatment in pneumococcal meningitis (PM), and daptomycin, a non-bacteriolytic antibiotic preventing pathogen-associated molecular pattern (PAMP) release.

**Results:**

Adjunctive inhibition of MRP14 or HMGB1 reduced mortality in mice with PM. This effect was lost when the two anti-DAMP agents were given simultaneously, possibly due to excessive immunosuppression. Combining anti-PAMP (daptomycin) and anti-DAMP treatments did not produce synergistic results; instead, the anti-DAMP treatment alone was sufficient and superior. The combination of anti-HMGB1 with dexamethasone did not diminish the effect of the former.

**Conclusions:**

DAMP inhibition possesses good potential as an adjuvant treatment approach in PM, as it improves clinical outcome and can be given together with the standard adjuvant dexamethasone without drug effect loss in experimental PM.

## Background

Pneumococcal meningitis (PM) is the most common form of bacterial meningitis in Europe and the USA [1]. It is still among the major causes of death from infectious diseases in adults worldwide, and survivors often suffer from brain damage and residual neurological deficits [2]. Extensive neutrophilic inflammation following pneumococcal infection of the meninges contributes substantially to brain damage [3]. Meningeal inflammation is triggered upon recognition of pathogen-associated molecular patterns (PAMP), liberated during pneumococcal autolysis, by pattern recognition receptors expressed on and in resident immune cells. As a consequence, neutrophils are recruited to the cerebrospinal fluid (CSF) [4, 5] and release all their antimicrobial weapons (like strong oxidants and proteolytic enzymes) into the extracellular space. The neutrophil-derived toxins, together with bacterial toxins (like pneumolysin and hydrogen peroxide), can cause stress and damage to host cells [6, 7]. The injured cells release then endogenous alarm signals (so-called danger-associated molecular patterns, DAMPs; [8, 9]) that sustain the immune reaction and drive forward brain damage even after CSF sterilization by antibiotics [10]. Such alarm signals are members of the S100 calgranulin family, especially myeloid-related protein MPR14, and high mobility group box 1 protein (HMGB1) [11, 12].

HMGB1 is a ubiquitously expressed nuclear protein that stabilizes nucleosome formation and regulates transcription [13]. In inflammation, it is secreted by immune cells after stimulation or can be released from dying cells [14, 15]. Once in the extracellular space, it induces and enhances cytokine synthesis [12], promotes chemotaxis and accumulation of granulocytes [16], and inhibits phagocytosis of apoptotic leukocytes [17]. It can exert its functions through both Toll-like receptor and the receptor for advanced glycation end products (RAGE) activation [18, 19]. Myeloid-related protein 14 (MRP14), in turn, is expressed and secreted mainly in myeloid cells in response to microbial components and inflammatory cytokines but can also be released from necrotic cells [20, 21]. Extracellular MRP14 can bind to pattern recognition receptors (PRRs), such as Toll-like receptor 4 (TLR4), and their interaction leads to the production of pro-inflammatory cytokines [22]. In vitro, MRP14 was also reported to support chemotaxis, migration, and antimicrobial activity of neutrophils and to have direct antimicrobial activity against pathogens [23–26].

We have previously shown that large amounts of MRP14 and HMGB1 are secreted in the CSF of patients and mice during acute PM and blockade of their activity by paquinimod, a MRP14 inhibitor, and ethyl pyruvate (REPS), and HMGB1 box A protein, HMGB1 inhibitors, was beneficial in experimental murine pneumococcal meningitis [27, 28]. Therefore, they may represent promising targets for adjuvant therapies in PM. Here, we evaluated the therapeutic efficacy of paquinimod and an anti-HMGB1 antibody, given alone or in combination, as adjuvant agents to antimicrobial treatment in a mouse model of PM and compared them to other adjuvant agents like dexamethasone and daptomycin.

## Methods

### Ethics statement

This study was performed in accordance with the recommendations in the National Institutes of Health Guide for the Care and Use of Laboratory Animals. The study protocol was approved by the local committee on the ethics of animal experiments (permit number 55.2-1-54-2531-31-09 and -78-12).

### Mouse model of pneumococcal meningitis

A well-characterized mouse model of PM was used in this study [29, 30]. Briefly, adult male C57BL/6 mice aged 8–16 weeks were clinically examined and scored. Clinical scoring consisted of a beam balancing test, a postural reflex test, and the presence of piloerection, seizures, or reduced vigilance [31]. In healthy animals, the score is 0 points; 13 points are attributed to terminally ill animals that have to be euthanized due to ethics within the observation period. Additionally, an open field test (as described previously [32]) and temperature measurements were carried out. After clinical scoring, meningitis was induced by intracisternal injection of 10^5^ colony-forming units of *S*. *pneumoniae* type 2 (D39 strain) under short-term anesthesia induced by isoflurane. Mice injected with equal volume of PBS served as negative controls (*n* = 8). In total, 100 mice were used in this study; two of them had to be euthanized immediately after intracisternal injection due to clinical signs of brain stem injury caused by the injection procedure. At 21 h after infection, all 98 mice achieved disease scores between 4 and 9 and were randomly allocated to the different i.p. treatment groups which were as follows: antibiotic therapy with 100 mg/kg ceftriaxone in combination with 100 μg anti-HMGB1 chicken polyclonal antibody (SHINO-TEST Corporation, *n* = 8), 10 mg/kg paquinimod (Active Biotech, *n* = 8), anti-HMGB1 + paquinimod (*n* = 6), 0.5 mg/kg dexamethasone (every 8 h, *n* = 9), anti-HMGB1 + dexamethasone (*n* = 8), 12 mg/kg daptomycin (*n* = 6), or anti-HMGB1 + daptomycin (*n* = 8). If not otherwise stated, treatments were given once per day. Administration of ceftriaxone alone (*n* = 12) served as positive control, while adjuvant injection with anti-HMGB1 isotype antibodies (*n* = 12) or DMSO (paquinimod vehicle, *n* = 9) were used as placebo-treated controls. Adjunctive treatments were injected 5 min before ceftriaxone injection. All treatments were applied immediately after one another. At the time when treatment is started, MRP14 and HMGB1 are conceivably present in the CSF since previous Western blot and ELISA experiments showed that MRP14 and HMGB1 are released into the CSF within the first 18 to 24 h post-infection in murine pneumococcal meningitis [28, 29]. Moreover, MRP14 and HMGB1 were detected in CSF samples withdrawn from patients with pneumococcal meningitis at the time of admission to the hospital [28, 29]. Twenty-four hours after therapy, animals were blindly clinically evaluated again. Then, mice were anesthetized with ketamine/xylazine, and a catheter was placed into the cisterna magna. CSF samples were withdrawn for the determination of CSF leukocyte counts and the intracranial pressure was measured. Subsequently, blood samples were obtained by transcardial puncture for the assessment of bacterial titers and total leukocyte counts. After deep anesthesia induced by thiopental, mice were perfused with ice-cold phosphate-buffered saline containing heparin. Brains were removed and frozen immediately. The experiment was performed in batches of 8 mice, with 1–2 mice randomly allocated to one of the different treatment groups, so that for every treatment group, the procedure was repeated at least thrice.

### Determination of bacterial titers in the blood and brain

Cerebella were dissected and homogenized in sterile saline. Blood samples and cerebellar homogenates were diluted serially in sterile saline, plated on blood agar plates, and cultured for 24 h.

### Analysis of cerebral bleeding

Mice brains were cut in a frontal plane into 30-μm-thick sections using a cryotome. Beginning from the anterior parts of the lateral ventricles (BREGMA 1.3 mm), every 10 sections (300 μm in total), a photograph was taken with a digital camera throughout the ventricle system to assess the number and total area of cerebral bleeding. Nine frames in total were photographed, covering a 2.7-mm interval in rostral-dorsal direction (up to BREGMA − 1.4 mm). Per frame, the number of blood spots was counted and the area of each blood spot was measured (Image J tool, NIH). From the data acquired, the total number and total area of blood spots per brain were calculated and compared.

### Statistical analysis

The principal statistical test was one-way ANOVA, followed by Student-Newman-Keuls post hoc testing. Differences were considered significant at *p* < 0.05. Data are displayed as means. Statistics was conducted with Prism, Graph Pad software.

## Results

**Fig. 1:**
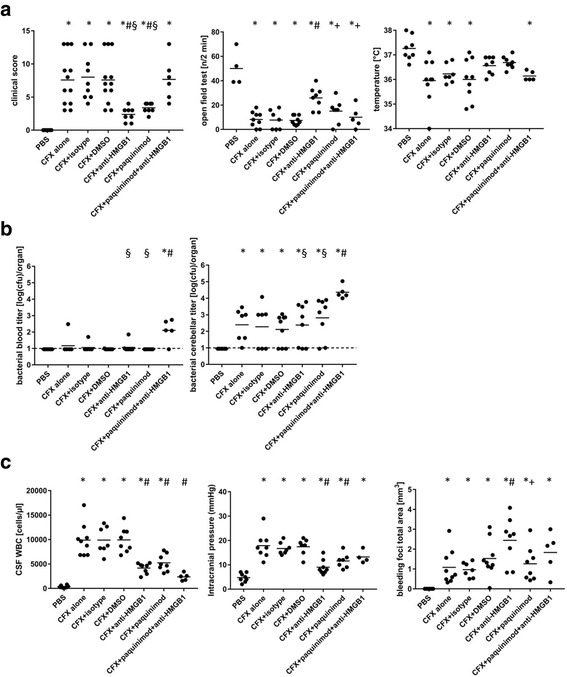
**a**–**c** Effects of paquinimod and anti-HMGB1 antibodies as adjuvants in the treatment of pneumococcal meningitis (PM). All infected mice were treated 21 h after infection with ceftriaxone (CFX) except for mice injected with PBS (which served as negative control). DMSO (vehicle for paquinimod) and isotype antibodies were used as placebo controls. Lumbar puncture and collection of clinical parameters (**a**), bacterial titers (**b**), and pathophysiological parameters (**c**) were performed 24 h after the start of treatment. The line within each group of points represents the mean of the group. Statistics were conducted using one-way ANOVA and Student-Newman-Keuls post hoc test. For the blood and cerebellar titer, the dotted line represents the lower detection limit at log(cfu) = 1. *p < 0.05, compared to PBS; #*p* < 0.05 compared to positive controls (CFX alone, CFX + isotype and CFX + DMSO); §*p* < 0.05 compared to CFX + paquinimod + anti-HMGB1; +*p* < 0.05 compared to CFX + anti-HMGB1

**Fig. 2:**
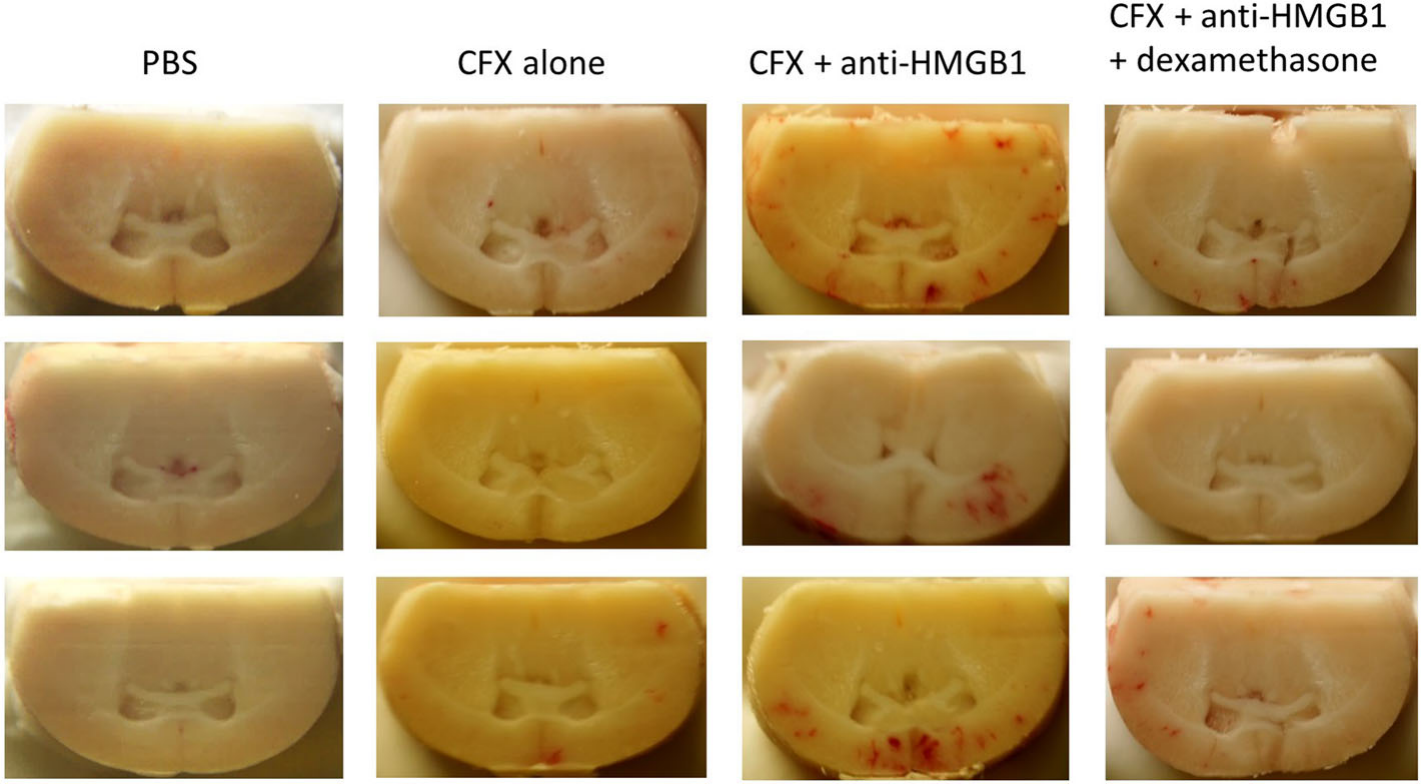
Comparison of the extent of cerebral bleeding after various treatments in pneumococcal meningitis (PM). Beginning from the anterior parts of the lateral ventricles, nine serial sections of brains were photographed with a digital camera in 0.3-mm intervals throughout the ventricle system. Per frame, the number of blood spots was counted and the area of each blood spot was measured. Depicted are representative examples of the following treatment groups: PBS (negative control), ceftriaxone (CFX) alone (positive control), anti-HMGB1 and anti-HMGB1 + dexamethasone

In a first experimental series, we tested the efficacy of paquinimod, anti-HMGB1 antibody, and a combination of both as adjuvant therapies in our experimental PM mouse model. Treatment was started 21 h after intracisternal pneumococcal infection. Positive controls received only ceftriaxone with or without placebo which was a DMSO solution and/or isotype control antibodies. (Fig. 1). Mice that were injected intracisternally with PBS instead of *S*. *pneumoniae* served as negative controls. The overall clinical outcome determined by a clinical score was better in mice treated adjuvantly with paquinimod or anti-HMGB1 antibodies, however not in those treated with their combination (Fig. 1a). This was also reflected in the mortality rates among the different treatment groups: in each adjuvant monotherapy group, all mice survived, whereas the mortality rates in the positive control groups and the adjuvant combination therapy group were 25, 25%, 22.2, and 16.7%, respectively (not significant). In other clinical parameters, anti-HMGB1 led to a significantly better performance in the open field test in comparison to all other groups, while paquinimod and the combination of anti-HMGB1 and paquinimod did not produce significantly better results than the control groups (Fig. 1a). Considering body temperature, anti-HMGB1 and paquinimod (mono) therapy were associated with milder meningitis-associated hypothermia than that found in positive control groups, while the combination of anti-HMGB1 and paquinimod showed body temperatures equal to these control groups. One possible explanation for the worse clinical outcome in anti-HMGB1 plus paquinimod-treated mice could be the significantly lower bacterial elimination from both the blood and the CNS, as compared to all other infected groups (Fig. 1b). This, in turn, might be linked to the observed dramatic reduction in CSF pleocytosis (Fig. 1c). The latter was also detectable in mice treated with anti-HMGB1 antibodies or paquinimod alone, however to a lesser degree (reduction by 58 and 47%, respectively, versus 76% in the combination group). Of note, the reduction in intracranial pressure (ICP) observed after combined treatment with anti-HMGB1 and paquinimod was less pronounced than that found after single administration of these drugs (Fig. 1c). Strikingly, while anti-HMGB1 performed overall better compared to paquinimod or the combination of both, it led to an increase in the total cerebral bleeding area (Fig. 2).

Since the adjuvant monotherapies with paquinimod and anti-HMGB1 were clearly superior to the combination therapy and anti-HMGB1 fared clinically slightly better than paquinimod, we focused in our next experiments on counteracting HMGB1. We addressed the question whether adjuvant anti-HMGB1 therapy is still effective when given (hours) after the start of antibiotic therapy. For this, infected mice were treated with ceftriaxone 21 h after infection, while 3 h later, mice received anti-HMGB1 antibodies. All positive effects of anti-HMGB1 that were seen in the previous experiments were not seen any more when anti-HMGB1 was given 3 h after antibiotics. For instance, clinical score and leukocyte count were doubled in the 3 h-post-treatment group (data not shown). These findings suggest that a timely administration is required to produce the best possible protective effect.

**Fig. 3:**
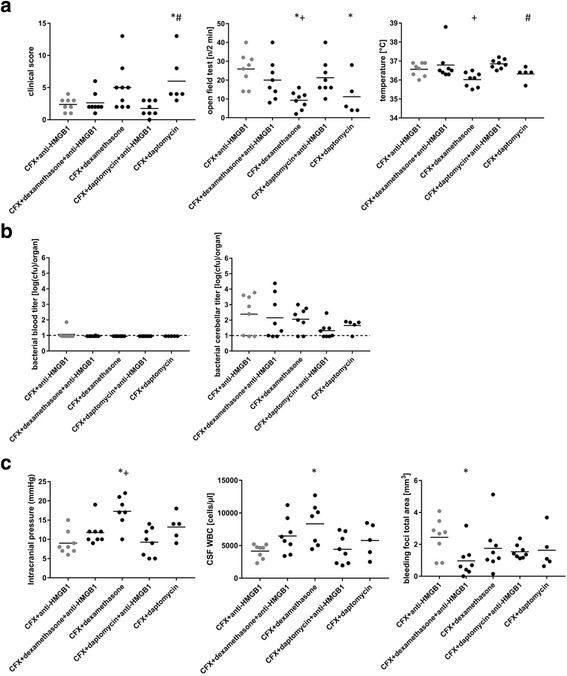
**a**–**c** Effects of combined administration of anti-HMGB1 antibodies and dexamethasone or daptomycin in the adjuvant therapy of pneumococcal meningitis (PM). All infected mice were treated 21 h after infection with ceftriaxone; isotype antibodies were used as placebo control. Lumbar puncture and collection of clinical parameters (**a**), bacterial titers (**b**), and pathophysiological (**c**) parameters were done 24 h after the start of therapy. The line within each group of points represents the mean of the group. Gray bars represent results already shown in Fig. 1. The treatment groups in gray were compared to either CFX + dexamethasone and CFX + anti-HMGB1 + dexamethasone or CFX + daptomycin and CFX + anti-HMGB1 + daptomycin. Statistics were conducted using one-way ANOVA and Student-Newman-Keuls post hoc test. For the blood and cerebellar titer, the dotted line represents the lower detection limit at log(cfu) = 1. *p < 0.05 compared to CFX + anti-HMGB1; +*p* < 0.05 compared to CFX + anti-HMGB1 + dexamethasone; #*p* < 0.05 compared to CFX + anti-HMGB1 + daptomycin

Currently, dexamethasone is the only adjuvant with proven effectiveness in adult patients with pneumococcal meningitis in countries with a high level of medical care but its effectiveness is far from being perfect. Thus, the impact of adding anti-HMGB1 to dexamethasone on the course of PM was assessed. In detail, we wanted to know whether both substances act in a synergistic immunosuppressive manner or whether dexamethasone co-application attenuates the protective effects of anti-HMGB1, since it has been shown to interfere with drug penetration to the CNS [33]. Dexamethasone monotherapy resulted in a slightly improved clinical status (as evidenced by significantly lower clinical score values) compared to untreated or placebo-treated controls (data not shown). When compared with mice treated with dexamethasone alone as standard adjunctive treatment, mice that received adjunctive anti-HMGB1 performed significantly better in the open field test and exhibited lower CSF leukocyte counts as well as ICP values (Fig. 3a–c). Co-administration of anti-HMGB1 and dexamethasone was comparably effective in improving the disease course as the anti-HMGB1-monotherapy, as indicated by similar clinical score values, motor activities, body temperatures, ICP values, and CSF leukocyte numbers (Fig. 3a, c). Interestingly, the co-administration of dexamethasone and anti-HMGB1 was associated with a significant reduction of the extent of cerebral bleeding as seen in mice who received adjunctive anti-HMGB1 only (Figs. 2 and 3c). Altogether, combining the two agents had the similar outcome as adjunctive anti-HMGB1 by itself, implying that dexamethasone does not interfere with the beneficial effects of anti-HMGB1.

Beta-lactam antibiotics, like ceftriaxone, which belong to the standard therapeutic regimen in PM trigger the autolytic degradation of pneumococcal cell walls, resulting in an abrupt release of PAMPs into the CSF, thus boosting the inflammatory host reaction. Non-bacteriolytic antibiotics like daptomycin and rifampicin have been proven beneficial in an infant rat and a rabbit model of PM [34, 35]. Therefore, we asked whether combining an “anti-PAMP” agent (the non-bacterioloytic antibiotic daptomycin) with a DAMP antagonist (the anti-HMGB1 antibody) could produce synergistic protective effects in our PM model. The combination of daptomycin and anti-HMGB1 led to a clinical outcome similar to that observed after adjunctive therapy with daptomycin or anti-HMGB1 alone (Fig. 3a). Bacterial blood titers were comparable in all three adjunctive treatment conditions (Fig. 3b) and equally low compared to untreated controls (data not shown), while cerebellar titers tended to be lower in the combination therapy group (not significant). There were also no between-group differences with regard to ICP values and leukocyte counts in CSF (Fig. 3c). Of note, daptomycin co-application was capable of dampening the adverse (side) effect of anti-HMGB1 on the meningitis-associated increase in cerebral bleeding (Fig. 3c). Overall, concurrent administration of anti-HMGB1 and daptomycin did not show synergistic effects in PM, suggesting that PAMP and DAMP release represents serial events (but not parallel pathways) in the pathophysiology in PM.

## Discussion

Pneumococcal meningitis is still considered a lethal infectious disease worldwide despite advances in therapy, due to extensive brain damage caused by sustained immune response even after the bacteria are eliminated [1]. We have postulated that the preservation of the immune reaction is attributed to the release of DAMPs by injured immune and neuronal cells even long after CSF sterilization of [27, 28]. Here, we tested if paquinimod, a known specific MRP14 inhibitor, and an anti-HMGB1 antibody have a potential as adjuvant therapies in PM. We showed (i) that anti-DAMP treatment had an additional protective effect in PM to the standard antibiotic treatment. Strikingly, (ii) this effect was lost when combining the two anti-DAMP agents. (iii) Combining the anti-HMGB1 treatment with dexamethasone, the standard adjuvant treatment of pneumococcal meningitis, neither improved nor diminished the effect of the former. Finally, (iv) combining anti-PAMP (daptomycin) and anti-DAMP treatment did not produce synergistic results; instead, the anti-DAMP treatment alone was sufficient and superior.

We had previously identified MRP14 and HMGB1 as two DAMPs playing a significant role in the pathobiology of PM and had shown that antagonizing them may be a promising therapeutic approach [27, 28]. In previous studies, we used ethyl pyruvate (REPS) and HMGB1 box A protein [27]. Although both agents have been shown to inhibit HMGB1 function, they were not ideal, as the former is an unspecific inhibitor of HMGB1 release, while the latter is a reversible competitive antagonist of extracellular HMGB1 that displaces HMGB1 binding to cells and thus cannot prevent HMGB1 function entirely [36, 37]. Here, we opted for an anti-HMGB1 antibody overcoming mentioned obstacles. In line with the previous data, anti-HMGB1 therapy proved clinically and pathologically protective in our PM model. Interestingly, in contrast to ethyl pyruvate and box A, anti-HMGB1 led to an increased extent of bleeding. Since the isotype antibody did not produce the same results, this adverse effect is suggestively the result of the specific interaction of anti-HMGB1 with its target rather than an antibody effect. However, the mechanism behind this side effect remains unknown and should be examined further in future studies. Paquinimod administration delivered similar protective results as we have previously observed [28]. When we co-administered paquinimod and anti-HMGB1 in order to find out whether this approach could lead to enhanced effectiveness, we strikingly observed a negation of the positive results of their individual administration. A possible explanation might be the significantly higher bacterial titers in the blood and brain observed in the combination therapy group. Insufficient bacterial clearance is known to negatively affect the clinical outcome [38]. The lower bacterial clearance could be associated with the lower leukocyte counts in CSF [39], suggesting the need of maintaining a certain threshold of immune reaction that can assist antibiotic therapy in pneumococcal elimination.

Since anti-HMGB1 performed slightly better than paquinimod, we concentrated our studies on the former. Addressing the question of the time point of the administration, we observed a considerable attenuation of the beneficial clinical effect when anti-HMGB1 was given 3 h after antibiotic treatment. This implicates that, even though HMGB1 is considered a relatively late inflammatory mediator and reaches high levels in the CSF in advanced disease stages [27, 40], inhibiting its function timely with the beginning of antibiotics is the essential way to prevent a prolongation of the immune response and brain damage. Early administration concurrent or immediately prior to antibiotics has already been shown for dexamethasone to have the best possible effect [41, 42] and comprises the current recommendation for the standard PM treatment [43]. Since then, all adjuvant approaches tested in animal models or clinical trials have adopted the concurrent treatment as with dexamethasone, like daptomycin or rifampicin [34, 35].

Various adjuvant therapeutic approaches aimed at dampening the inflammatory reaction and protecting the brain from injury have been evaluated in animal PM models. Among them, a prominent group of agents are non-bacteriolytic antibiotics like daptomycin and rifampicin. In previous studies, those antibiotics led to a similar bacteria clearance in CSF as ceftriaxone, though causing less inflammation and brain damage by killing bacteria without cell lysis and thus preventing the release of PAMPs [34, 35]. Since PAMPs and DAMPs are the motors of inflammation in PM, we combined anti-PAMP (daptomycin) and anti-DAMP (anti-HMGB1) treatment to investigate if blocking both pathways produces synergistical effects. Interestingly, the combination therapy had no additional effects on clinical parameters than HMGB1 alone. These results suggest that PAMP and DAMP signaling are not parallel pathways but rather serial events, and blocking the DAMP signaling pathway alone is pivotal and sufficient to reduce brain damage and improve clinical outcome of PM. In previous studies, where non-bacteriolytic antibiotics had a substantial positive effect on clinical outcome, they were given simultaneously to up to 1 h before ceftriaxone [35]. The application regimen of drugs (e.g., the timing of the administration of individual drugs) can possibly influence their therapeutic efficacy. That could also be the case when combining anti-PAMP and anti-DAMP treatments in bacterial meningitis. Here, we opted for a simultaneous treatment regimen having in mind the potential clinical translation. In the clinical setting, when bacterial meningitis is suspected, empiric antimicrobial treatment has to be given promptly and should not be delayed while awaiting (first) therapeutic effects of potential adjunctive drugs. This is because early antibiotic treatment is associated with better outcome [44, 45].

Dexamethasone is to date the only adjuvant with proven additional clinical benefit and thus the drug of choice for adjunctive treatment of PM [41, 43]. One well-known problem for dexamethasone is interference with drug penetration into the CNS, resulting in lower CSF levels of various substances, such as vancomycin [46] and daptomycin [33], which in turn can lead to a lower bacterial clearance and therapeutic failure. Since adjunctive dexamethasone has been advocated for adult patients with PM (in high-income countries) by IDSA and ESCMID guidelines [47, 48], every other potential agent for adjunctive treatment should not interact with it in a detrimental manner. Therefore, we examined the impact of anti-HMGB1 in addition to adjunctive dexamethasone in our PM model. The protective activity of adjunctive anti-HMGB1 treatment was not diminished when combined with dexamethasone, albeit the two agents did not act synergistically, indicating the possibility of a co-administration of dexamethasone and anti-HMGB1 antibodies in PM. Even more relevantly, the only drawback observed in the treatment with anti-HMGB1, the increase in the number and total area of bleeding foci, was counteracted by its combination with dexamethasone, probably due to improvement of the blood-brain-barrier [49].

## Conclusions

In summary, anti-HMGB1 possesses good potential as an adjuvant therapeutic strategy in PM, as it improves clinical outcome and can be given together with the standard treatment of antibiotics and dexamethasone. Thus, it may deserve consideration for future clinical studies.

## Abbreviations

CNS: Central nervous system
CSF: Cerebrospinal fluid
DAMP: Danger-associated molecular patterns
HMGB1: High mobility group box 1 protein
ICP: Intracranial pressure
MRP14: Myeloid-related protein 14
PAMP: Pathogen-associated molecular patterns
PM: Pneumococcal meningitis
PRR: Pattern recognition receptors
